# Project to Improve the Transcription of Clinical Order Information into a Radiology Information System

**DOI:** 10.51894/001c.6936

**Published:** 2018-09-26

**Authors:** Michael J. Mills, John X. Nguyen, Ben Himelhoch, Abdelouahid Souala, Anthony Khashola, Sumita Joseph, Phillip Rathousky, Roger Gonda, Michael C.Y. Juan

**Affiliations:** 1 Providence-Providence Park Hospital/Michigan State University College of Human Medicine Diagnostic Radiology Residency Program, Southfield, MI; 2 3 Michigan State University College of Human Medicine Southeast Michigan Campus, Southfield, MI; 3 Michigan State University College of Human Medicine Southeast Michigan Campus, Southfield, MI; 4 Michigan State University College of Osteopathic Medicine Diagnostic Radiology Residency Program State Wide Campus – Garden City Hospital, East Lansing, MI

**Keywords:** quality improvement, radiology information system, imaging order transcription

## Abstract

**CONTEXT:**

Inaccurate and incomplete imaging order information presented to interpreting radiologists is a persistent problem in many radiology settings. Computerized Physician Order Entry processes in clinic-based settings are often inconsistent, and radiology transcription clerks continue to play a critical role in transmitting accurate content and information from referring physician orders to the radiology information system. (RIS) The purpose of this quality improvement project was to a) identify common transcription areas of deficient RIS imaging order information and b) test outcomes from an intervention to improve the content and concordance of transcribed patient information entered into the RIS.

**METHODS:**

A random convenience sample of 500 outpatient radiographic orders were categorized according to degree and quality of concordance between the transcribed patient information documented in the RIS and the corresponding original imaging order information. During Phase I, the authors used a root-cause analysis to determine the possible etiologies for discordance between the information in original imaging orders and the information transcribed into the RIS. The intervention that was delivered included a short education session with radiology transcription clerks with placement reminder posters at transcription workstations. During Phase 2, a second random sample was obtained following the intervention, with data collection and analyses replicating the process from Phase I. A set of inferential comparisons were conducted using chi-square tests to examine for statistical significance.

**RESULTS:**

There was an overall 44% decrease in transcription discordance (p < 0.001), and the number of cases with perfectly concordant RIS order indication documentations increased by 21% (p < 0.001). A total of 34% of transcriptions from Phase I were partially discordant due to an inadequate imaging study indication, compared to 15% during Phase II (p < 0.001). There was also a 22% increase in the number of completely concordant transcriptions free of grammatical errors (p < 0.001).

**CONCLUSIONS:**

A short education session with radiology transcription clerks along with placement of reminder posters may significantly improve both the concordance and quality of transcribed imaging order information presented to interpreting radiologists using the RIS.

## INTRODUCTION

The transcription of inaccurate and incomplete radiologic imaging order information remains a persistent problem in many settings.^1,2^ The entry of accurate information concerning the indications for an imaging order (i.e., reason radiologic procedure was ordered) and adequate patient history can impact the quality of reports, frequently affecting patient safety and imposing billing problems.^1–6^ Prior studies have revealed that as many as 30% of imaging order requisitions can lack adequate clinical order indications, and up to 24% can lack vital patient information for proper image interpretation.^3,5^

Radiology transcription clerks remain important personnel responsible for recognizing both grammatical and content imaging order errors.^1^ Transcription clerks also play a critical role in determining which information to transfer from the original imaging order into radiology information systems (RIS).^1^

Computerized physician order entry (CPOE) is a major functionality that could potentially mitigate errors made by radiology transcription clerks. However, the literature reveals that by 2004 only 10% of US institutions had fully implemented a CPOE system.^7,8^ By 2015, this proportion had only increased to 15.7%.^8,9^ Numerous radiologist authors have discussed the importance and necessity of improving the imaging order information presented to the radiologist through use of CPOE software systems.^4,5,10^

However, there appear to have been few studies examining the transcription of imaging order information to a RIS after implementation of CPOE.^8^ The systematic testing of interventions to improve transcription of order imaging information to date has also been rare.^8^ In one study, however, educating transcription clerks, supplemented by a checklist, was shown to significantly improve, from 46.4% to 62.8% “perfect” concordance, of the information presented to interpreting radiologists.^1^

At the authors’ community-based institution, CPOE has been implemented for hospital radiology imaging orders with direct population of order information into the RIS. At the time of this project, this was not the case for the institution’s clinic-based orders. When patients presented for clinic imaging, they provided a registration clerk with a hard copy imaging order requisition from the referring physician. These forms could either be standardized fillable forms or written narrative note prescriptions. A radiology transcription clerk then manually transcribed the imaging order information into the RIS. Picture archiving and communication system (PACS), Merge RadSuite^11^ was also used to convey imaging order indications, the imaging modalities ordered, and other pertinent patient information to the radiologist who later interpreted the imaging results. An overview of the general process at the authors’ healthcare system is depicted in Figure 1.

**Figure:**
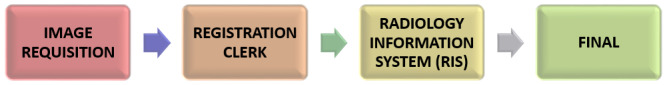
Figure 1 Flow of information at Providence-Providence Park Hospital from patient presentation with image requisition to final report.

Similar to other settings, the authors had ongoing key problems related to the transcription process in this setting including the entry of inaccurate or incomplete information from the image requisition form into the RIS, grammatical transcription errors, and an inadequate amount of clinical patient history.^1^ These errors ranged from minor grammatical errors and misspellings, to inappropriate abbreviations, missing patient information, and some absent information or random typographical errors.

These types of errors can potentially lead to compromised patient safety and diminished efficiency for the radiology practice, which in turn can be associated with unnecessary loss of time and aberrant radiologic reports being sent back to the referring physician.^1–5^ Finally, billing and coding staff use this information for revenue recovery, and incomplete transcription information can also delay system reimbursements.^1^

### Purpose of Project

Our two-phase quality improvement (QI) project was conducted to: a) identify common transcription areas of deficient RIS imaging order information (Phase I) and b) test outcomes from an intervention to improve the content and concordance of transcribed patient information entered into the RIS (Phase II).

## METHODS

After project approval was obtained from the Providence-Providence Park Hospital Institutional Review Board, Phase I of the project was conducted with the goal of examining the extent of discordant information found between imaging orders and information entered into the RIS. The authors utilized retrospective chart review from both the PACS and RIS systems to identify the most common sources of transcription errors. Data from a random sample of 500 subjects were gathered from the imaging orders, RIS documentation and the PACS. The subjects included in this first phase had received clinic-ordered imaging studies from September 1 - October 1, 2016. Studies reviewed included radiographs, computed tomography, magnetic resonance imaging, and nuclear medicine studies.

The image order documentation, consisting of either a form generated by a computer at the referring physician’s office, or a written prescription from the ordering physician, was scanned directly into the PACS system and reviewed by authors BH, AS, AK, and MCYJ under the supervision of authors MJM and JXN. The clinical information from these documents, as previously transcribed to the RIS for review by the interpreting physician, was also reviewed by the same authors. This information was cross-referenced by these same authors to assess the degree and quality of concordance between the image order documentation and the RIS presented to the interpreting physician. More specialized imaging studies ordered in the hospital setting and/or completed through the interventional and fluoroscopy radiology service were excluded from the analytic sample.

### Phase I: Historical Review of RIS Order Information

Upon their initial review of sample records, it was clear to the authors that the greatest source of incomplete or inaccurate RIS order information was related to transcription clerk errors. For this study, the authors used the general three-category approach of DiRoberto, Lehto and Baccei (2016) to gauge concordance levels in RIS data between the two study phases.^1^ Imaging studies were categorized as “concordant” if the information matched verbatim and as “partially concordant” if the RIS did not contain all of the information present in the imaging order form. Imaging studies were categorized as “discordant” if a substantial amount of necessary information was missing in the RIS, or if the RIS clearly contained incorrect information or typographical errors. Classification decisions were based on clinical judgement and agreed upon by all authors.

Concordant and partially concordant imaging orders were also assessed for quality of concordance. Quality of concordance was categorized as “highest” if transcribed information matched the clinical order verbatim, contained a complete and informative patient history, and was free of any grammatical errors. The quality of concordance was categorized as “high” if transcribed information was concordant but contained grammatical errors (e.g., incorrect capitalization, non-standard abbreviation, misspellings or misused punctuation). The quality of concordance was categorized as “low” if information in the RIS was partially concordant with the actual imaging order but failed to provide a complete or accurate patient history. Also categorized as low concordance were records with significant grammatical errors and/or omissions that contained ambiguities that could not be readily interpreted by the RIS auditor.

Following Phase I, a meeting was conducted with four administrators, the authors, and the department chair. A root-cause analysis, shown in Figure 2, was used to determine the possible etiologies for transcription discordance between the imaging order information and the information transcribed into the RIS.

**Figure:**
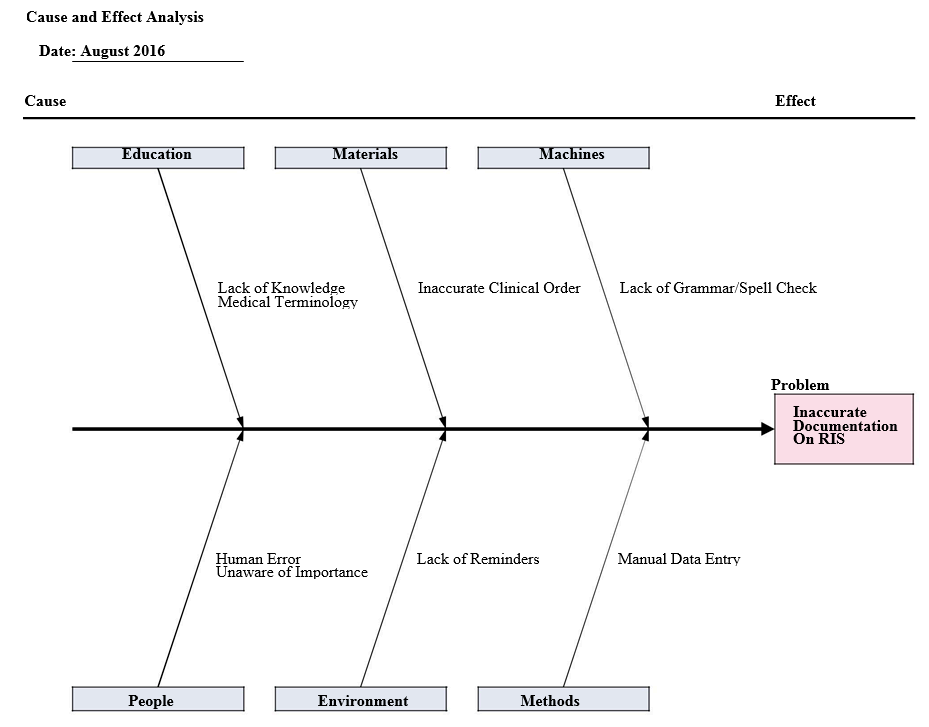
Figure 2 Root-cause analysis of authors’ transcription improvement initiative.

### Project Intervention

Based on the authors’ Phase I findings, transcription process errors were chosen as the target of intervention. The intervention included an initial staff meeting in June 2017 to inform radiologists of the QI project. A detailed email was also routed to all radiology transcription clerks, emphasizing their ongoing attention to accurate and complete transcription of imaging order information. Examples of correct and full transcriptions were presented (Appendix 1).

In addition, an 8 x 11 inch poster including a checklist for verbatim transcription of content, grammatical checking, and spell checking was placed at each transcription workstation (Appendix 2). A reminder was also included as part of regular mid-month department meetings and emails.

Following the intervention, Phase II of the study entailed the same data collection process as Phase I. The subjects included in Phase II of the study had received clinic-based imaging orders between June 1 to July 1, 2017. Again, more specialized imaging studies completed in the hospital setting and/or through the interventional and fluoroscopy radiology services were excluded from the analytic sample. Data from both the image requisition and RIS documents were again extracted on the same variables and cross-referenced by the data collector to assess their degree of transcription concordance. Inferential comparisons utilizing chi-square statistical testing were utilized. Two PhD-prepared researchers (see acknowledgements section) at the first author’s healthcare system used SPSS™ version 24.0 statistical software ^12^ to conduct analyses.

## RESULTS

A total of 266 (60%) of Phase I imaging order documentation records were found to be perfectly concordant, meaning that the content provided for the indication and clinical history was identical between the imaging order and RIS documentation. Phase II demonstrated 374 (81%) perfect concordance, a 21% post-intervention increase (p < 0.001). A total of 121 (34%) of RIS documents from Phase I were partially discordant, meaning the order indication and patient history information was partially but inadequately transcribed compared to 68 (15%) during Phase II (p < 0.001). A total of 24 (6.0%) Phase I RIS documents were discordant, meaning substantial information was missing, compared to 19 (4%) during Phase II (p = 0.271). Overall, the number of partially or completely discordant documents decreased by 44% after intervention (p < 0.001) (Figure 3).

**Figure:**
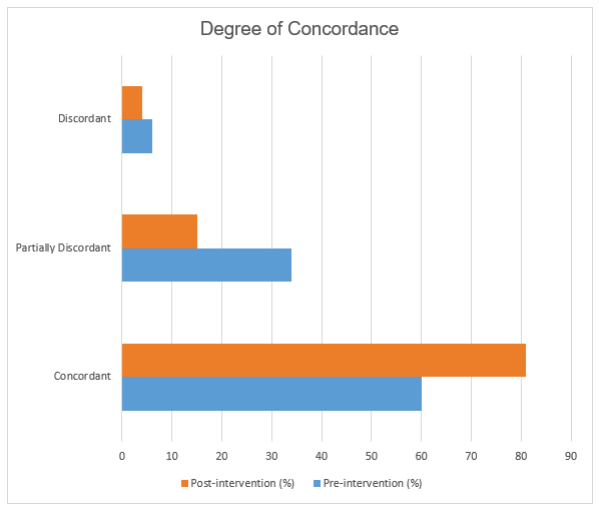
Figure 3 Graph Demonstrating the Degree of Concordance Between Information Appearing on Original Imaging Order Compared to Information Transcribed to RIS Before and After Intervention.

Regarding overall levels of transcription concordance, there was a 22% (169 pre-intervention and 265 post-intervention) increase in the number of transcriptions that demonstrated the highest level of concordance (p < 0.001) (Figure 4). For the remainder of sample cases, there were substantial grammatical errors made during imaging order transcription into the RIS. These problems could be attributable to either the imaging order information having been entered manually, with lack of an electronic spell check function within the RIS documentation software, or variable medical terminology training/experience among radiology transcriptionists.

**Figure:**
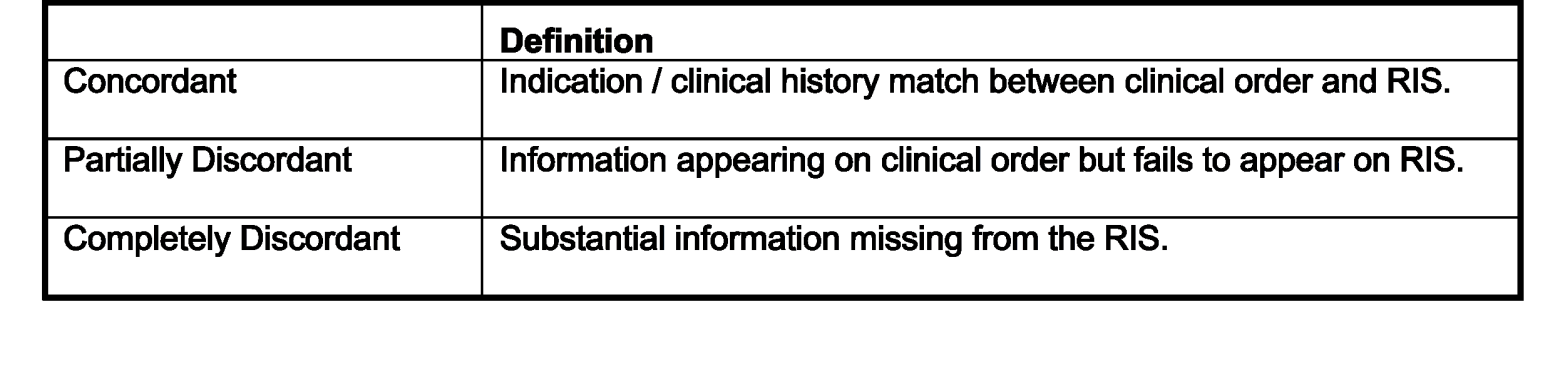
Figure 4 Concordance Classification Framework for Degree of Concordance

**Figure:**
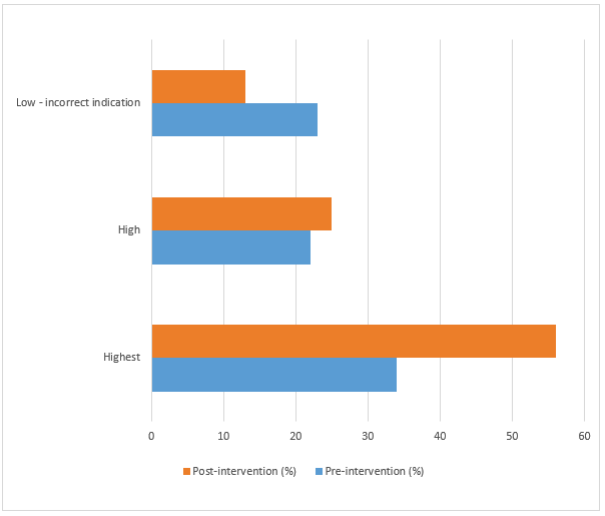
Figure 5 Quality of Concordance

**Figure:**
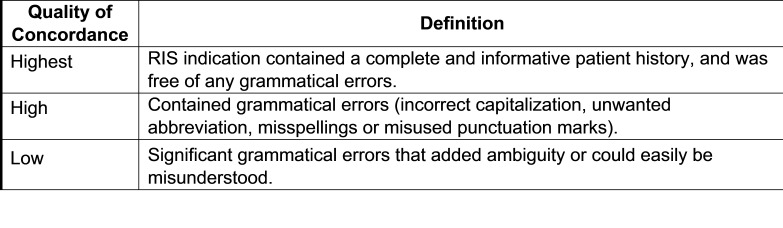
Figure 6 Concordance Quality Classification Framework

Observed concordance errors included omissions of specific clinical information provided by the referring physician, for example the clinical order said “shoulder pain” while the transcribed information on the RIS said “pain.” There were also inappropriate abbreviations used, such as “PE” substituted for “pulmonary embolism” which could also be interpreted as “pleural effusion.” Other RIS forms omitted relevant information that was present on the original imaging order, such as the site/side of injury, symptoms provided by the referring physician, or their specific concerns regarding the suspected clinical pathology. Other spelling and grammatical errors were observed, and in some cases, nonsensical information was transcribed such as random typographical errors or repetition of a single letter such as “aaaaaaaaaa.”

Although the imaging order information was somewhat concordant in a portion of these cases, any discrepancies still could have had implications related to radiologic efficiency and effectiveness, especially for radiologists’ final interpretive report dictations. While any imperfect information could have been be edited in the radiologists’ dictation notes, this may have added an unnecessary time-consuming step. In cases where radiologists failed to note or correct auto-populated errors, an unclear or incomplete final report could have been added to both patients’ permanent medical records and referring physicians.

## DISCUSSION

The authors’ Phase I data revealed the scope of errors that had occurred during the process of transcribing information from original imaging order into the RIS. 40% of sample records were discordant with their corresponding imaging order indication information, demonstrating substantial differences between what referring physicians had communicated about patients versus what information was immediately available to the radiologist interpreting the image results. Similar transcription error rates have been shown in previous studies. For example, a similar 2016 study showed the number of perfectly concordant RIS indication information increased from 232 (46.4%) to 314 (62.8%) after the implementation of a similar intervention.^1^ Similarly, the number of partially concordant matches decreased from 162 (32.4%) to 114 (22.8%).^1^

In cases of discordant information, the interpreting radiologist may need to investigate patients’ documented histories and order indications through review of the patients’ medical records and/or direct communication with the referring physician. Inadequate imaging order indication information can also potentially delay reimbursement and consume time utilized to obtain missing information.

Our QI project intervention was shown to improve significantly both the concordance and quality of transcribed RIS information. Although there was one Phase I case in which the imaging study performed was inappropriate due to an incorrect study indication on the RIS, there were no such cases in Phase II. This finding suggests that although rare, there can be potential patient safety improvements derived from these types of QI interventions.

### Project Limitations

Our smaller-scale project was conducted at a single Michigan healthcare system and we only measured outcomes during one month following the intervention. While a statistically significant improvement in transcription concordance levels was measured, the sustainability of these improvements remains unclear. A future project would be beneficial to investigate the longer-term effects of the intervention, and whether periodic repeated intervention reminders could extend the sustainability of these achieved improvements.

Although our QI intervention primarily focused on the RIS transcription process as a primary source of errors, several other potential sources were identified during our root cause analysis. Our intervention was not specifically focused on reducing cases in which the referring physician had failed to provide the radiologist an adequate amount of patient history and/or information concerning imaging order indications. This is another potential source of errors that may become increasingly common as CPOE becomes more widely implemented.^1^ The increased programming of electronic spell check function into RIS software programs may provide another potential source of improvement.^2,4^

## CONCLUSIONS

These QI project results indicate that a short educational session for radiology transcription clerks, along with placement of reminder posters, could significantly improve both the concordance and quality of transcribed information presented to interpreting radiologists on RIS documents. Future large-scale controlled samples are required to more fully examine the numerous factors likely to influence the many complex steps entailed in contemporary RIS information flow processes across our nation’s imaging departments and clinics.

## DISCLOSURES

Overall study findings were presented on a poster at the 3rd Annual Michigan Summit on Quality Improvement and Patient Safety in Troy, MI on June 1, 2018, and were accepted for presentation at the Radiological Society of North America 104^th^ Scientific Assembly and Annual Meeting in Chicago, Il, November, 2018.

### Conflict of Interest

The authors declare no conflict of interest.

## Figures and Tables

**Figure:**
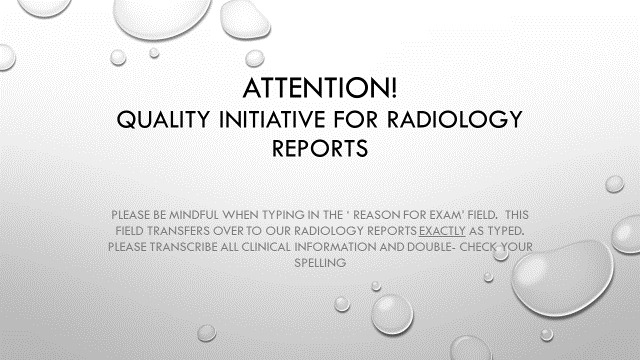
Appendix 2 Transcription Clerk Workstation Poster

## Appendix 1

### Intervention Email

“We will be starting a new quality initiative regarding improving the accuracy of radiology requisitions. We are asking associates to pay special attention to accurately transcribing the clinical order to the radiology requisition form. Please note that the information placed in the”reason for exam” is transferred to the dictated final report that is sent to the ordering physician. Particular attention should be paid to transcription of the order as it is written (Reason for Exam/Indication) to avoid spelling / grammatical errors. Should you notice any spelling / grammatical errors in the Cerner “reason for exam” field, please correct them prior to submitting the order in DOE or prior to completing the order in exam management. We will be collecting data to assess how much improvement we were able to achieve. Periodic reminders will be sent over this time period, and we will share the results once they are compiled. We appreciate your dedication to quality and your participation in this initiative.”
